# The Effect of Rev-erbα Agonist SR9011 on the Immune Response and Cell Metabolism of Microglia

**DOI:** 10.3389/fimmu.2020.550145

**Published:** 2020-09-25

**Authors:** Samantha E. C. Wolff, Xiao-Lan Wang, Han Jiao, Jia Sun, Andries Kalsbeek, Chun-Xia Yi, Yuanqing Gao

**Affiliations:** ^1^Department of Endocrinology and Metabolism, Amsterdam University Medical Centers, University of Amsterdam, Amsterdam, Netherlands; ^2^Laboratory of Endocrinology, Amsterdam University Medical Centers, Amsterdam Gastroenterology & Metabolism, University of Amsterdam, Amsterdam, Netherlands; ^3^Key Laboratory of Cardiovascular and Cerebrovascular Medicine, School of Pharmacy, Nanjing Medical University, Nanjing, China; ^4^Laboratoire de Neuroscience Cognitives et Adaptatives, Université de Strasbourg, Strasbourg, France; ^5^Netherlands Institute for Neuroscience, Royal Netherlands Academy of Arts and Sciences, Amsterdam, Netherlands

**Keywords:** clock genes, Innate immunity, microglia, immunometabolism, neuroinflammation, cytokines, phagocytosis

## Abstract

Microglia are the immune cells of the brain. Hyperactivation of microglia contributes to the pathology of metabolic and neuroinflammatory diseases. Evidence has emerged that links the circadian clock, cellular metabolism, and immune activity in microglia. Rev-erb nuclear receptors are known for their regulatory role in both the molecular clock and cell metabolism, and have recently been found to play an important role in neuroinflammation. The Rev-erbα agonist SR9011 disrupts circadian rhythm by altering intracellular clock machinery. However, the exact role of Rev-erbα in microglial immunometabolism remains to be elucidated. In the current study, we explored whether SR9011 also had such a detrimental impact on microglial immunometabolic functions. Primary microglia were isolated from 1–3 days old Sprague-Dawley rat pups. The expression of clock genes, cytokines and metabolic genes was evaluated using RT-PCR and rhythmic expression was analyzed. Phagocytic activity was determined by the uptake capacity of fluorescent microspheres. Mitochondria function was evaluated by measuring oxygen consumption rate and extracellular acidification rate. We found that key cytokines and metabolic genes are rhythmically expressed in microglia. SR9011 disturbed rhythmic expression of clock genes in microglia. Pro-inflammatory cytokine expression was attenuated by SR9011 during an immune challenge by TNFα, while expression of the anti-inflammatory cytokine *Il10* was stimulated. Moreover, SR9011 decreased phagocytic activity, mitochondrial respiration, ATP production, and metabolic gene expression. Our study highlights the link between the intrinsic clock and immunometabolism of microglia. We show that Rev-erbα is implicated in both metabolic homeostasis and the inflammatory responses in microglia, which has important implications for the treatment of metabolic and neuroinflammatory diseases.

## Introduction

The circadian brain clock orchestrates physiological and metabolic processes to prepare the body for environmental changes and to optimize energy metabolism (1). Cells in peripheral tissues, including metabolic tissues such as liver, pancreas, and kidney, also display rhythms in activity that are regulated by an intrinsic clock machinery. Since metabolic events are tightly regulated by the circadian clock, disruptions of the circadian timing system can result in metabolic dysfunctions and contribute to obesity and type 2 diabetes (2–10). Thus, the intracellular clock system is tightly involved in the control of metabolic rhythms that are essential for metabolic homeostasis.

The nuclear receptor reverse viral erythroblastosis oncogene product alpha (Rev-erbα) plays a crucial role in both the molecular clock of the circadian timing system and the regulation of metabolism. Rev-erbα stabilizes circadian rhythms by inhibiting the expression of the core clock genes brain and muscle ARNT-Like 1 (*Bmal1)* and circadian locomotor output cycles kaput (*Clock*) (11–13). The known endogenous ligand of Rev-erbα is heme, which plays an important role in mitochondrial respiration, and is required for the repressive activity of Rev-erbα/β on target genes (14–17). Additionally, nuclear receptors are sensors for dietary lipids and lipid-soluble hormones, thus rhythmic expression of Rev-erbα/β is also involved in regulating metabolic processes in a circadian manner (18, 19). Intriguingly, a recent Rev-erbα knock-out study proposed an important role for Rev-erbα in regulating neuroinflammation (20). In previous research, Rev-erbα agonists (GSK4112 and SR9011), and antagonists (SR8278) have provided us with insights about the function of Rev-erbα (21–24). For example, systemic administration of the Rev-erbα agonist SR9011 altered clock genes expression and disrupted the circadian behavior of mice leading to loss of locomotor activity during the active phase (dark phase), while vehicle administration caused no disruption (23). These results show that Rev-erb nuclear receptors have profound effects on circadian rhythm, metabolism and neuroinflammation, and possibly are eligible targets for treating metabolic diseases.

Recent research has demonstrated that a high fat, high sugar diet is associated with chronic activation of microglia, also known as microgliosis, which contributes to disturbed energy homeostasis and diet-induced obesity (DIO) (25–29). Additionally, our group recently reported that microglia activity shows a clear day/night rhythm when animals are fed a regular diet, but this rhythm is disrupted in DIO (30, 31). These findings suggest that the daily microglial rhythm is important for its normal activity and disturbance may result in metabolic diseases. However, little is known about the mechanism behind the intrinsic clock and the function of microglia. There is also a complex interplay between metabolic processes and immune responses, known as immunometabolism, in which metabolic reprogramming underlies the inflammatory state of microglia (32, 33). Therefore, considering the important role of Rev-erbα in the molecular clock machinery, neuroinflammation, and metabolism, in the current study we used the Rev-erbα agonist SR9011 to investigate the role of Rev-erbα in microglial immunometabolism.

## Materials and Methods

### Primary Microglia Culture

Primary microglia cultures were prepared from brains of newborn, male Sprague Dawley rat pups (1–3 days old; Nanjing Medical University, China). After removal of the meninges, the brains were dissected and homogenized. Tissue lysates were centrifuged and suspended in Dulbecco’s modified Eagle’s medium (DMEM; Gibco, United States; C11885500BT) containing 1 *g*/L D-Glucose, L-Glutamine, 110 mg/L sodium pyruvate, and supplemented with 10% Fetal Bovine Serum (FBS; Gibco, United States; 10099141) and 1% Penicillin/Streptomycin (Gibco, United States; 15140122). Mixed glia cells were plated at a density of one brain per T75 flask or three brains per T175 flask, and incubated at 37°C in a humid atmosphere with 5% CO2. Culture medium was refreshed every 3 days. After the astrocyte layer reached confluency (8–14 days), the microglial cells were collected by shaking the flasks 150 rpm/min for 1 h at 37°C. Cells were seeded in 96-well plates (30k–50k cells/well) for the 3-(4,5-dimethylthiazol-2-yl)-5-(3-carboxymethoxyphenyl)-2-(4-sulfophenyl)-2H-tetrazolium (MTS) assay, 12-well plates (250k–300k cells/well) for qPCR experiments, or coverslipped in a 24-well plate (100k–150k cells/well) for phagocytosis experiments, or the Seahorse XF96 Cell Culture Microplate (Agilent, United States; 50k cells/well) for Seahorse experiments. For palmitic acid study, the palmitic – BSA solution was prepared 5:1 molar ratio palmitate: BSA in Krebs ringer buffer. All plates were coated with poly-L-lysine hydrobromide (MDBio, China). Dexamethasone (Sigma-Aldrich, United States; D4902), SR9011 (Sigma-Aldrich, United States; SML2067), DMSO (MDBio, China; D015), Fatty acids free BSA (Sigma A7030), Sodium Palmitate (Sigma-Aldrich, United States; P9767), TNFα (Peprotech, United States; 315-01A), and 0.1% BSA (Beyotime, China; ST023) were used to treat the cells during the experiments.

### Cell Viability Assay

Cell viability was determined with the CellTiter 96 Aqueous assay (Promega, United States; G3582). Cells were treated with dexamethasone for 2 h, followed by a 24-h treatment with 5 μM SR9011 (or DMSO for control). Afterward, MTS solution was added followed by incubation for 3 h at 37°C, and the absorbance at 490 nm was measured on a SpectraMax M2 microplate reader (Molecular Devices).

### Real-Time PCR

For gene expression analysis, total RNA was extracted from primary microglia using the RNAeasy^TM^ kit (Beyotime, China; R0026) according to the manufacturer’s protocol. RNA concentration was measured with the NanoDrop Onec Spectrophotometer (Thermo Scientific, United States) and reverse transcribed into cDNA using the HiScript II qRT Supermix (Vazyme Biotech; R222-01). Gene expression levels of *Bmal1, Clock, Nr1d1 (Rev-erb*α*), Per2, Cry1, Il1*β, *Il4 Il6, Il10, Tnf*α, *Ccl2, Gm-csf, Tgf*β, *CD36, CD68, Cpt1, Pdk1, Hk2, Fasn, Glut5*, and *Hprt* (housekeeping gene; see Supplementary Table 1 for primer sequences) were measured using real-time quantitative PCR on a QuantStudio 5 (Applied Biosystems Thermo Scientific), using the AceQ qPCR SYBR Green Master Mix (Vazyme Biotech; Q131-02). Gene expression levels were normalized to the housekeeping gene. Primers were designed using the Basic Local Alignment Search Tool (BLAST) from the National Center for Biotechnology Information (NCBI) and purchased from GeneRay Biotech.

### Phagocytosis Assay

After treatment with SR9011 or DMSO for 12 h, microglia were incubated with 0.05% fluorescent latex microspheres (Sigma, United States; L1030-1 ml) in DMEM containing 0.25% FBS for 1 h. Subsequently, the cells were fixed with 4% paraformaldehyde for 30 min and a fluorescence staining of microglia was performed with Rabbit Anti-Iba1 (Wako, Japan; 019-19741) and the nuclei were counterstained with 4’,6-diamidino-2-phenylindole (DAPI; Bioprox, France). Images were captured with an Olympus BX53F microscope equipped with an Olympus U-HGLGPS light source, and analyzed with ImageJ software (version 1.48). The relative fluorescent intensity of the beads was determined by dividing the total fluorescent intensity of the beads by the number of DAPI-stained nuclei.

### Cellular Bioenergetics

Microglia were treated with 5 μM SR9011 or DMSO for 4 h prior to placement in the Seahorse XFe96 Analyzer (Agilent, United States). During the run, oxygen consumption rate (OCR), and extracellular acidification rate (ECAR) were measured and the wells were injected with modulators from the Agilent Seahorse XF Mito Stress Kit to determine parameters of mitochondrial function. After measuring basal levels of OCR and ECAR, oligomycin was injected to block ATP synthase, which decreases mitochondrial respiration. The second injection was carbonyl cyanide-4 (trifluoromethoxy) phenylhydrazone (FCCP), which disrupts the mitochondrial membrane potential and maximizes oxygen consumption. The third injection was a mixture of rotenone and antimycin A, which block complex I and III (respectively) and shut down mitochondrial respiration. Lastly, 2-deoxy-D-glucose (2-DG) was added to inhibit glycolysis, leading to a decrease in ECAR. Cellular respiration, mitochondrial respiration, cellular acidification, maximum substrate utilization, and maximum glycolytic capacity were calculated as previously described (34). ATP production was calculated from mitochondrial respiration by using a phosphate/oxygen ratio of 2.3 (35). After the measurements, cells were fixed with 4% paraformaldehyde and attached cells were quantified using a crystal violet assay by measuring absorbance at 590 nm with a SpectraMax M2 microplate reader (Molecular Devices). Data obtained from the Seahorse XF96 Analyzer was normalized to cell quantity per well.

### Statistical Analyses

All results are expressed as mean ± SEM. Statistical analysis was performed using Graph-Pad PRISM (version 7.00), ImageJ software (version 1.48), and JTK_Cycle software (36). Two-way ANOVA and Bonferroni’s *post hoc* test was used to assess the SR9011 effects on clock genes within 24 h after synchronization. Unpaired *t*-tests were used to evaluate the differences between DMSO and SR9011 groups in the rest of the study. ImageJ software was used for the analysis of phagocytosis assays. JTK_Cycle software (36) was used to identify rhythmic components in circadian PCR data.

## Results

### Rhythmic Expression of Cytokines and Metabolic Genes in Microglia

**FIGURE 1:**
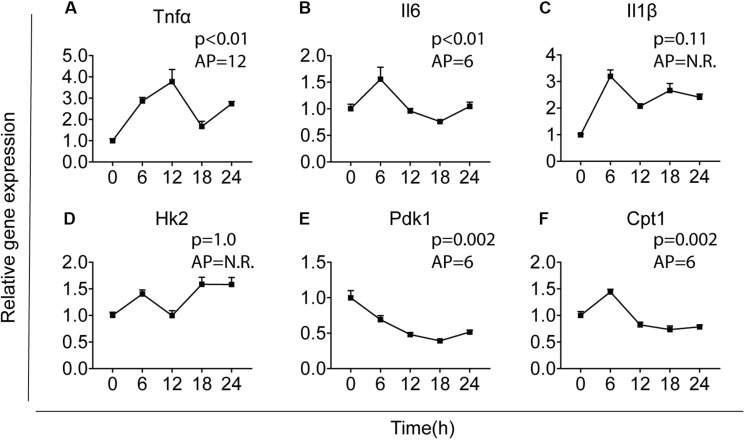
Major cytokines and metabolic genes are rhythmically expressed in microglia. Relative gene expression of major cytokines and metabolic genes was measured in primary microglia. After synchronization by dexamethasone for 2 h, cells were cultured and harvested at the indicated post-synchronization time points (T; *n* = 6 per group, per time point). Rhythmic expression is signified by a *p*-value of less than 0.05 and can be found for *Tnf* **(A)**, *Il6* **(B)**, *Pdk1* **(E)**, and *Cpt1* **(F)**. The expression of *Il1* **(C)** and *Hk2* **(D)** was not rhythmic. The acrophase of the curve is not relevant (N.R.) if data does not fit a curve. Data are presented as means ± SEM and statistical significance was determined using JTK_Cycle software.

The intrinsic clock machinery is present in microglia with clock genes being rhythmically expressed (30, 37). It has been well documented that cytokines and metabolic genes exhibit rhythmic expression (23, 38). To confirm whether cytokines and metabolic genes were also temporally regulated in primary microglia, we investigated the expression of three key pro-inflammatory cytokines – interleukin-1 beta (*Il1*β), interleukin-6 (*Il6*), and tumor necrosis factor-alpha (*Tnf*α), as well as three important metabolic genes – carnitine palmitoyltransferase 1 (*Cpt1*), hexokinase 2 (*Hk2*), and pyruvate dehydrogenase kinase 1 (*Pdk1*). Primary microglia were synchronized by 10 nM dexamethasone for 2 h as reported before (39) and cultured for 0, 6, 12, 18, or 24 h before being harvested. Gene expression of the cytokines and metabolic genes was determined over these time points [time post-synchronization, from now on referred to as “Time (T)” in hours] and rhythmicity was analyzed by JTK_Cycle software (Figure 1). *Tnf*α, *Il6, Pdk1*, and *Cpt1* showed a clear rhythmic expression, while *Il1*β and *Hk2* were not rhythmically expressed (Figure 1). The acrophase of the curves was estimated at T6, for *Il6, Pdk1* and *Cpt1*, and T12 for *Tnf*α. These data show that *Il6, Tnf*α, *Pdk1*, and *Cpt1* expression are likely orchestrated by the circadian timing system in primary microglia.

### SR9011 Disrupts Clock Gene Rhythmicity in Microglia

**FIGURE 2:**
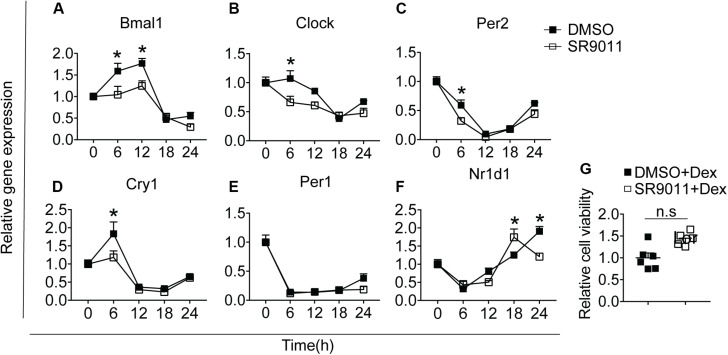
SR9011 disrupts clock gene expression in microglia. Rhythmic and relative gene expression of clock genes in primary microglia. Cells were treated with dexamethasone for 2 h followed by SR9011 or DMSO and were harvested at the indicated time points (*n* = 4 per group, per time point). **(A–F)** Clock gene expression is altered by SR9011 treatment compared to DMSO (control). SR9011 disrupted the rhythmic expression of *Bmal1* and *Clock*, and caused a shift in the acrophase of *Per2* rhythm, as analyzed by JTK_Cycle. **(G)** Cell viability of primary microglia was not affected by the 24-h SR9011 treatment with dexamethasone. Data are presented as means ± SEM. *p* < 0.05* vs. DMSO group was determined using Two-way ANOVA followed by Bonferroni’s *post hoc* test and multiple comparison.

We first determined whether SR9011 enhances Rev-erbα activity and disrupts the clock machinery in primary microglia. For this, we performed a dose-response study with 1, 5, 10, and 50 μM SR9011 (data not shown) and decided to use 5 μM for this study. To test the efficiency of SR9011, we first analyzed the effects of SR9011 on Rev-erb targets *Serpine1*, *Cyp7a1*, and *Srebf1* and found significant reductions in their expression compared to control, which confirms that SR9011 acts through Rev-erb (Supplementary Figure 1). When using SR9011 as a pretreatment before synchronization with dexamethasone, we found that the effect of SR9011 pretreatment did not persist after removing SR9011 from the culture (Supplementary Figure 2 and Supplementary Table 2), contrary to the result from Nakazato et al. (39). Therefore, the following design was used in this study: primary rat microglia were exposed to dexamethasone for 2 h to synchronize the cells, followed by treatment with. 5 μm SR9011 or DMSO for 0, 6, 12, 18, or 24 h. Rhythmic expression of *Bmal1*, *Clock*, period 2 (*Per2)*, cryptochrome 1 (*Cry1)*, period 1 (*Per1)* and nuclear receptor subfamily 1 group d member 1 (*Nr1d1* or *Rev-erb*α) was found in primary microglia treated with DMSO, as evaluated with Two-way ANOVA (Table 1) and JTK_Cycle Software (Figure 2 and Table 2). SR9011 exerted an inhibitory effect on *Bmal1* and *Per2* expression, as analyzed by Two way ANOVA (Table 1). An interactive effect of SR9011 and Time was found on *Bmal1*, *Clock*, and *Nr1d1* (=*Rev-erb*α, see Table 1). The impact of SR9011 at each time point was analyzed by multiple comparison (Figures 2A–F). Notably, SR9011 disrupted the rhythmic expression of *Bmal1* and *Clock*, and caused a shift in the acrophase of *Per2* rhythm, as analyzed by JTK_Cycle (Table 2). SR9011 in combination with dexamethasone had no impact on cell viability (Figure 2G), indicating that a decrease in gene expression could not be attributed to cell death. These results show that SR9011 disrupted the rhythm of clock gene expression, pertaining to both amplitude and phase depending on the clock gene, which means that rhythmic expression of clock gene expression became non-rhythmic after adding SR9011. SR9011 had the strongest impact on *Bmal1* and *Clock*, while the effect on *Per2* and *Cry1* was less potent. This is probably due to the direct inhibitory effect of Rev-erbα on the BMAL1 and CLOCK complex, which indirectly influences *Per2* and *Cry1* expression.

**TABLE 1:** Two-way ANOVA assessment of effect of Time, SR9011, and Interaction in clock genes in primary microglia with SR9011.

**Genes**	**Two-way ANOVA analysis**		
	***p*-value**		
	**Interaction**	**Time**	**SR9011**
*Bmal1*	**0.0195**	**<0.0001**	**0.0370**
*Clock*	**0.0251**	**<0.0001**	0.0659
*Cry1*	0.1234	**<0.0001**	0.0907
*Per1*	0.5030	**<0.0001**	0.3939
*Per2*	0.0648	**<0.0001**	**0.0489**
*Nr1d1*	**0.0003**	**<0.0001**	0.4462

*SR9011, Time and Interaction effects were evaluated in primary microglia for clock genes after treated with DMSO or SR9011. SR9011 has significant impact on *Bmal1* and *Per2*, and has interaction effect with time on *Bmal1*, *Clock*, and *Nr1d1*.*

**TABLE 2:** JTK_Cycle analysis of clock genes in primary microglia with SR9011.

**Gene**	**DMSO**		**SR9011**	
	***P*-value**	**Acrophase**	***P*-value**	**Acrophase**
*Bmal1*	**0.0053**	9	0.0980	N.R.
*Clock*	**0.0293**	9	1	N.R.
*Cry1*	**<0.0001**	6	**0.0006**	6
*Per1*	**<0.0001**	0	**0.0293**	0
*Per2*	**<0.0001**	3	**<0.0001**	0
*Nr1d1*	**0.0364**	21	**<0.0001**	21

*Clock genes rhythmicity in microglia treated with SR9011 are analyzed by JTK_Cycle software. All the clock genes were rhythmically expressed in primary microglia treated with DMSO, signified by a *p*-value of less than 0.05. SR9011 disrupted the rhythmic expression of *Bmal1* and *Clock*. The acrophase of the curve is not relevant (N.R.) when data does not fit a curve.*

### SR9011 Alters Cytokine Expression in Microglia

**FIGURE 3:**
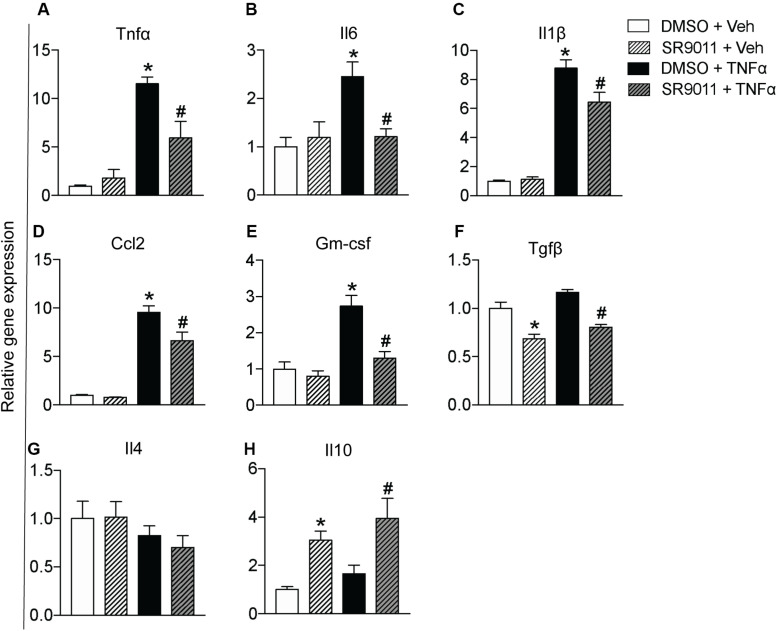
The effect of SR9011 on cytokine expression in microglia. Relative gene expression of cytokines in primary microglia treated with SR9011 or DMSO for 12 h followed by TNFα or BSA treatment for 12 h (*n* = 6 per group). This figure shows the effect of TNFα and SR9011 on pro-inflammatory cytokine expression **(A–E)**, regulatory cytokine expression **(F)**, and anti-inflammatory cytokine expression **(G–H)**. TNFα increases the gene expression of *Tnf*α, *Il6, Il1*β, *Ccl2*, and *Gm-csf*. SR9011 attenuates the expression of *Tnf*α, *Il6*, *Il1*β, *Ccl2*, and *Gm-csf* compared to treatment with TNFα alone. Conversely, SR9011 increases *Il10* expression after TNFα stimulation. Data are presented as means ± SEM and statistical significance was determined using Unpaired *t*-test in all experiments. *p* < 0.05* vs DMSO + BSA and *p* < 0.05^#^ vs DMSO + TNFα.

Cytokine secretion constitutes an essential part of microglial immune function. Rev-erbα has very recently been reported to regulate neuroinflammation (20). To evaluate the role of Rev-erbα in regulating cytokine expression, we stimulated the cells with TNFα to resemble an immune challenge and analyzed the effect of SR9011. Primary microglia were exposed to 5 μM SR9011 (or DMSO for control) for 12 h, followed by 100 ng/ml TNFα treatment (or BSA for control) for 12 h in combination with SR9011 or DMSO. TNFα significantly increased the expression of the pro-inflammatory cytokines tumor necrosis factor-alpha (*Tnf*α), interleukin-6 (*Il6)*, interleukin-1 beta (*Il1*β), and C-C Motif Chemokine Ligand (*Ccl2*; Figures 3A–E). TNFα also significantly increased the regulatory cytokine granulocyte-macrophage colony-stimulating factor (*Gm-csf*; Figure 3F). In all cases, the increase in gene expression by TNFα treatment was attenuated after treatment with SR9011. Different results were found for anti-inflammatory cytokines. TNFα had no effect on the regulatory cytokine transforming growth factor-beta (*Tgf*β) expression, however, SR9011 treatment decreased *Tgf*β expression (Figure 3G). No changes were found in interleukin-4 (*Il4)* expression (Figure 3H). Interestingly, while TNFα had no effect on the expression of anti-inflammatory cytokine interleukin-10 (*Il10)*, SR9011 stimulated the expression of *Il10* (Figure 3I). These results suggest that SR9011 attenuates the pro-inflammatory response in primary microglia in the context of an immune challenge, while stimulating the expression of the anti-inflammatory cytokine *Il10*.

### SR9011 Attenuated Palmitic Acid Induced Inflammatory Response in Microglia

**FIGURE 4:**
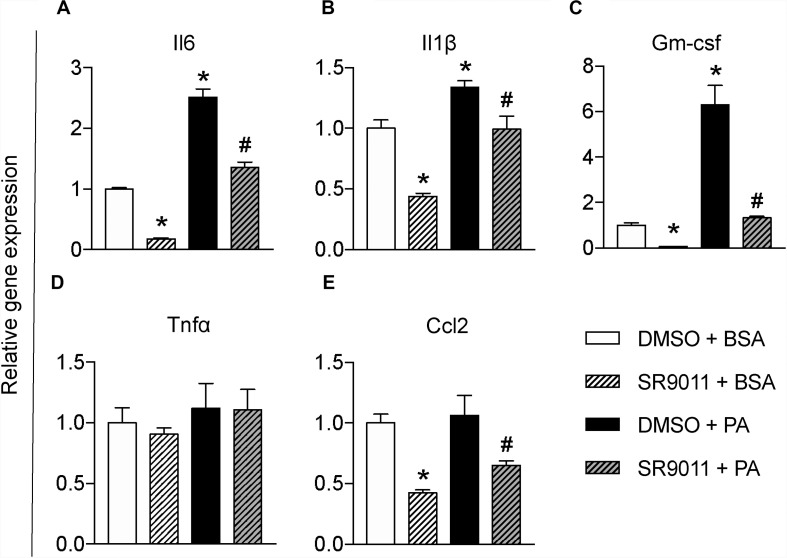
The effect of SR9011 on palmitic acid induced inflammatory responses in microglia. Relative gene expression of cytokines in primary microglia treated with SR9011 or DMSO for 12 h followed by 50 μM palmitic acid or BSA treatment for 12 h (*n* = 4 per group). Palmitic acid treatment increases *Il6* **(A)**, *Il1*β **(B)**, and *Gm-csf* **(C)**, which are inhibited by SR9011. Palmitic acid treatment has no profound effect on the expression of *Tnf*α **(D)** and *Ccl2* **(E)**, while SR9011 decreased *Ccl2* expression with and without palmitic acid treatment **(E)**. *p* < 0.05* vs DMSO + BSA and *p* < 0.05^#^ vs DMSO + PA.

To further test whether SR9011 has a potential therapeutic effect for overnutrition-induced neuroinflammation, we stimulated the microglia with palmitic acid (PA) to resemble a pro-inflammatory stimulus on high-fat diet feeding. Primary microglia were exposed to 5 μM SR9011 (or DMSO for control) for 12 h, followed by 50 μM palmitic acid (or BSA for control) for 12 h in combination with SR9011 or DMSO. Palmitic acid significantly increased the expression of pro-inflammatory cytokines *Il6* and *Il1*β. which were attenuated by SR9011 (Figures 4A,B). Palmitic acid also significantly stimulated *Gm-csf* expression, while SR9011 had a profound inhibitory effect on *Gm-csf* expression (Figure 4C). *Tnf*α and *Ccl2* expression was not enhanced by palmitic acid treatment (Figures 4D,E), which is a slightly different outcome compared to the TNFα stimulus. In addition, palmitic acid treatment exerted impacts on the expression of core clock genes *Nr1d1* and *Per1* after 12h incubation (Supplementary Figure 3), which indicates palmitic acid treatment might also influence circadian rhythms. This is consistent with our previous observation that circadian rhythms of microglia are disrupted in DIO animals (30). Thus, these data suggest that SR9011 attenuates the pro-inflammatory response in primary microglia upon palmitic acid treatment, which may be beneficial for DIO-induced neuroinflammation.

### SR9011 Decreases Phagocytosis in Microglia

**FIGURE 5:**
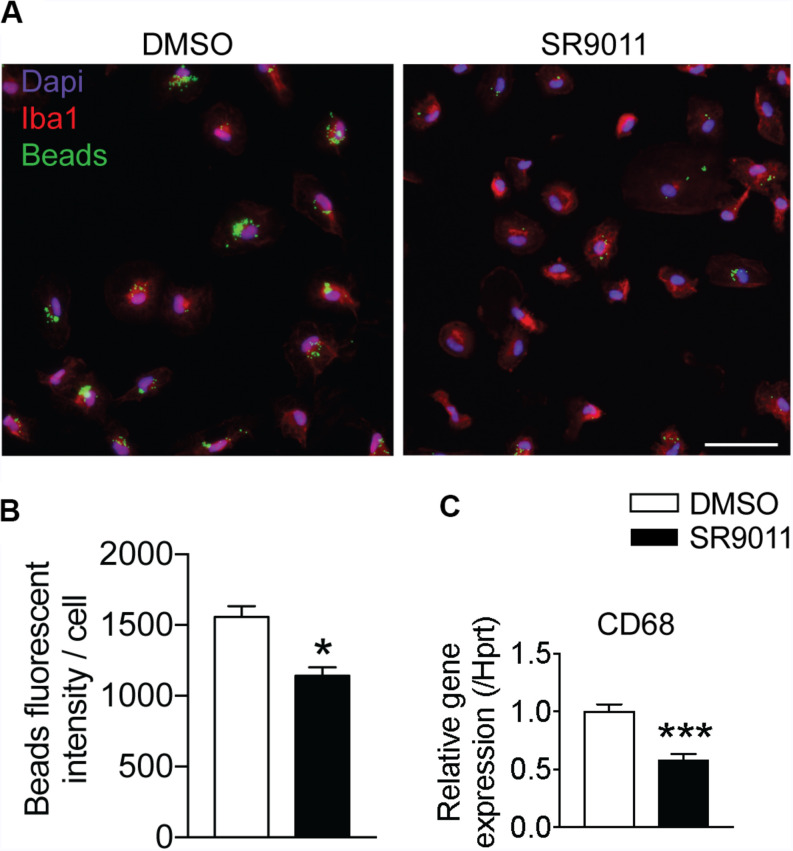
SR9011 decreased phagocytic activity in primary microglia. **(A)** The uptake of fluorescent beads by primary microglia treated with DMSO or SR9011. **(B)** Primary microglia treated with SR9011 exhibit decreased phagocytic activity (*n* = 15). **(C)** The expression of phagocytic marker *CD68* is decreased in primary microglia treated with SR9011 for 12 h (*n* = 6). Data are presented as means ± SEM and statistical significance was determined using Unpaired *t*-test in all experiments. *p* < 0.05* and *p* < 0.001*** vs. DMSO.

One of the main neuroprotective functions of microglia is phagocytosis, so we evaluated the effect of SR9011 on phagocytic activity. Primary microglia were treated with 5 μM SR9011 (or DMSO) for 12 h and subsequently exposed to 0.05% fluorescent beads for 1 h. A fluorescence staining was performed for IBA1 (microglia marker) and DAPI, after which the intensity of fluorescent beads per cell was determined (Figure 5A). The uptake of fluorescent beads was decreased in primary microglia treated with SR9011 (Figure 5B). Additionally, the expression of cluster of differentiation 68 (*CD68*), a phagocytic marker, was decreased in primary microglia that were starved for 6 h (0% FBS) and subsequently treated with 5 μM SR9011 for 12 h (Figure 5C). The purity of the cultures was tested by staining primary microglia with IBA1 and DAPI and a microglia purity of 98.81% was determined (data not shown). These results indicate that SR9011 decreases phagocytic activity, meaning that Rev-erbα plays an inhibitory role in regulating phagocytosis.

### SR9011 Inhibits Mitochondrial Respiration and Metabolic Gene Expression in Microglia

**FIGURE 6:**
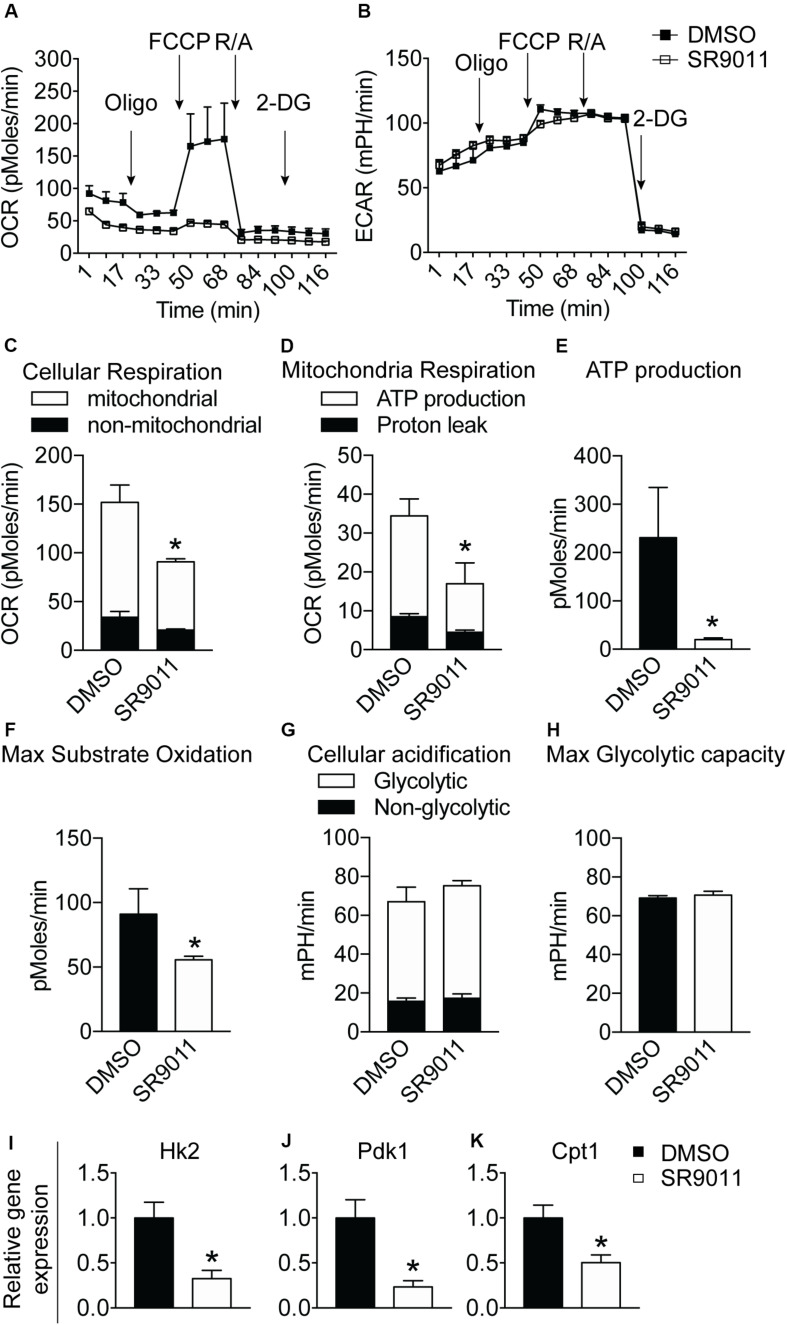
The effect of SR9011 on cellular metabolism in primary microglia. Cellular bioenergetics and metabolic gene expression in primary microglia after SR9011 treatment (*n* = 4 per group). **(A, B)** Overview of OCR **(A)** and ECAR **(B)** of primary microglia treated with DMSO or SR9011 for 12 h. Indicated are the times on which oligomycin, FCCP, rotenone/antimycin A (R/A) and 2DG were administered. **(C–H)** SR9011 decreased cellular respiration **(C)**, which was caused by a decline in ATP-linked mitochondrial respiration **(D)**. Consequently, ATP production was significantly decreased in response to SR9011 **(E)**. SR9011 also caused a decrease in maximum substrate oxidation **(F)**. No changes were found in cellular acidification **(G)** and maximum glycolytic capacity **(H)**. **(I–K)** SR9011 decreased the expression of metabolic genes *Hk2* **(I)**, *Pdk1* **(J)**, and *Cpt1* **(K)** in primary microglia treated by dexamethasone for 2 h and SR9011 for 6 h. Data are presented as means ± SEM and statistical significance was determined using Unpaired *t*-test in all experiments. *p* < 0.05*.

Major metabolic genes are expressed in a rhythmic manner in microglia, as shown in Figure 1. We evaluated metabolism after disrupting the intrinsic microglial clock with SR9011. Primary microglia were treated with 5 μM SR9011 (or DMSO) for 12 h after which cellular respiration and ECAR were measured using the Seahorse XFe96 Analyzer to evaluate mitochondrial respiration and glycolysis. The raw data of OCR and ECAR are shown in Figures 6A,B. Cellular respiration was significantly decreased by SR9011, which was mainly attributed to less ATP-linked mitochondrial respiration, while the H^+^ proton leak remained unchanged (Figures 6C,D). Consequently, ATP production was greatly reduced (Figure 6E). Furthermore, maximum substrate oxidation was decreased after SR9011 treatment (Figure 6F). No changes were found in cellular acidification (glycolysis) and maximum glycolytic capacity (Figures 6G,H). These results indicate that SR9011 decreases ATP production by inhibiting oxidative phosphorylation. This mitochondrial dysfunction is not compensated with glycolysis. Furthermore, SR9011 inhibits the expression of *Hk2*, *Pdk1*, and *Cpt1* (Figures 6I–K), which are key enzymes involved in substrate utilization by the citric acid cycle. Taken together, these results suggest an overall decrease in cellular metabolism caused by activation of Rev-erbα by SR9011. Thus, Rev-erbα is a potent inhibitor of cell metabolism in primary microglia.

## Discussion

The aim of the current study was to determine the role of Rev-erbα in microglial immunometabolism. We show that key cytokine and metabolic genes are rhythmically expressed in primary microglia. Activation of Rev-erbα with SR9011 disrupted the intrinsic microglial clock and attenuated the phagocytosis and the pro-inflammatory response. SR9011 also decreased mitochondrial respiration and metabolic gene expression in microglia. These findings shed new light on the link between the circadian clock, cell metabolism and immune function in microglia and identify Rev-erbα as a possible important therapeutic target for treatment of neuroinflammatory diseases.

The rhythmic expression of major cytokines and metabolic genes in primary microglia observed in the current study supports our previous idea that *in vivo* the intrinsic clock is coupled with cellular metabolism (30). Such rhythmicity of cytokines may have physiological relevance *in vivo* – we reported before that microglia activity displays a day and night difference and is accompanied by a change in TNFα expression in the medial basal hypothalamus, which in turn affected the mitochondria function of nearby neurons (31). A growing body of literature reports that an obesogenic diet is associated with constant activation of microglia, leading to chronic hypothalamic inflammation, which contributes to the pathology observed (26, 27, 31, 40). We also reported that microglial circadian rhythmicity is disrupted in DIO animals (27). In this study, we found that SR9011 prevents the microglia pro-inflammatory responses usually observed during an inflammatory challenge and an obesogenic metabolic challenge. The mechanism behind the metabolic improvements observed in DIO mice can partially be explained by the mitigating effect of SR9011 on hyperactive microglia, thereby attenuating inflammation. This finding can have major implications for reducing microgliosis caused by obesity or neuroinflammatory diseases. A better *ex vivo* experimental setting to mimic the *in vivo* situation under a chronic high-fat diet challenge would be helpful to further elucidate these mechanisms.

The current study examined the immune function of microglia upon SR9011-associated Rev-erbα activation. Similar topics have been discussed before. In studies on knock-out mice or mice pharmacologically administered with SR9011, microglia activation and *Il*6 expression was downregulated upon LPS exposure (39, 41). In another Rev-erbα deletion study, mice displayed microgliosis, increased CD68, and neuroinflammation in the hippocampus (20). Consistent with these results, we enhanced Rev-erbα activity with SR9011 and found a decreased pro-inflammatory response and phagocytosis. A recent study describes a regulatory role of Bmal1 in macrophage motility and phagocytosis (42), which may indicate a possible interplay between Rev-erb and Bmal1 in the regulation of phagocytosis in microglia – an interesting topic for future research. Additionally, SR9011 stimulated the expression of the anti-inflammatory cytokine *Il10*. This suggests Rev-erbα not only inhibits pro-inflammatory cytokines, but also promotes the anti-inflammatory response. Moreover, to more accurately mimic microglia’s immune response, we used TNFα as an innate immune challenge, which is more physiologically relevant compared to lipopolysaccharide (LPS) used in the studies mentioned above. These findings suggest that the molecular clock components Bmal1 and Rev-erbα are closely related to the microglial immune response, and that Rev-erbα activity can regulate the inflammatory state in microglia under physiological and pathological conditions.

Rev-erbα nuclear receptors were reported to regulate metabolic homeostasis in cancer cells, hepatic cells and hepatoma cells (17, 18, 43). The findings of the current study show that Rev-erbα nuclear receptors regulate cell metabolism in primary microglia, the innate immune cells, as evidenced by the decrease in mitochondrial respiration and ATP production, as well as the decreased expression of rate-limiting enzymes such as *Cpt1*, *Pdk1*, and *Hk2* in response to SR9011. Our study dissected how the Rev-erbα-associated clock machinery interacts with immunometabolism in microglia. It is generally assumed that immune function is tightly linked to cell metabolism due to the metabolic demand of an inflammatory response (44). One explanation for the decline in energy metabolism is that it is a consequential phenomenon caused by less energy demand due to the attenuated pro-inflammatory response. Another possibility is a direct effect of Rev-erbα on cellular metabolism. Rev-erbα nuclear receptors, when bound to their ligand, repress the transcription of not only clock genes like *Bmal1*, but also repress metabolic genes and are involved in mitochondrial respiration (14, 15, 45, 46). The inflammatory response of microglia also involves metabolic reprogramming, which also might explain the attenuation of pro-inflammatory cytokines (32, 33). Another speculation would be that the intrinsic molecular clock orchestrates both microglial metabolism and immune response, and that it can also change the activation state of microglia. The latter would mean that SR9011-induced enhanced Rev-erbα activity is associated with disturbed microglial circadian rhythms, which consequently changed the cellular metabolism and immune response. Indeed, the *in vivo* situation might be far more complicated, because it is unknown what occurs first in the development of metabolic disorders: the disrupted circadian rhythmicity, the alterations in intracellular metabolism, the changes in immune response, or any other unknown factors. In our opinion, the disrupted circadian rhythmicity is very likely to be the driving force of the other two. In addition, the appropriate immune response of microglia is required to defend the CNS under normal conditions, so attenuated pro-inflammatory responses might not be always beneficial for the body. Further research is needed to elucidate the causal relation among the intracellular clock, cellular metabolism and immune response in microglia under physiological condition and metabolic challenges. It is interesting to note that SR9009, a REV-ERB agonist similar to SR9011, has been reported to exert Rev-erbα independent side-effects on cell proliferation and metabolism (47). Whether SR9011 has the same side-effects is currently unknown, and requires further research.

Future research should also investigate the significance of the microglial intrinsic clock *in vivo.* It is likely that the exact consequences of disturbing the microglial clock depend on the brain regions involved. Microgliosis in the hypothalamus is associated with metabolic disorders, while microglia in other brain regions will have other functions, depending on the micro-environment. On the other hand, the role of Rev-erbα should also be investigated in other immune cells and their related immune-diseases.

## Conclusion

Our study demonstrates that disturbing circadian rhythmicity by SR9011-induced Rev-erbα activation attenuates pro-inflammatory cytokine expression and reduces cellular metabolism in microglia. In addition, expression of the anti-inflammatory cytokine *Il10* is stimulated, emphasizing the mitigating effect of Rev-erbα activation on the pro-inflammatory response. The findings of this study identify an interconnected role of Rev-erbα in the circadian rhythmicity, cell metabolism and inflammatory response of microglia and may have important implications for the employment of Rev-erbα as a potential therapeutic target for the treatment of neuroinflammatory diseases, however, further studies are required to achieve this goal.

## Data Availability Statement

The datasets presented in this study can be found in online repositories. The names of the repository/repositories and accession number(s) can be found in the article/Supplementary Material.

## Ethics Statement

The experiments were approved by the Animal Care and Use Committee of Nanjing Medical University, and the experiments were performed according to the Guide for the Care and Use of Laboratory Animals of China.

## Author Contributions

SW, JS, HJ, and YG performed the experiments. C-XY and YG designed the study. SW, X-LW, and AK analyzed the data and wrote the manuscript. All authors read and approved the final manuscript.

## Conflict of Interest

The authors declare that the research was conducted in the absence of any commercial or financial relationships that could be construed as a potential conflict of interest.
