# Supplementary material for: The Effect of Rev-erbα Agonist SR9011 on the Immune Response and Cell Metabolism of Microglia
Source: Front Immunol. 2020 Sep 25;11:550145. doi: 10.3389/fimmu.2020.550145 (PMC7546349; doi:10.3389/fimmu.2020.550145)
Supplement: Supplementary file 1 [file Data_Sheet_1.pdf]

The effect of Rev-erbα agonist SR9011 on the immune response and cell metabolism of microglia

Samantha E.C. Wolff[1,2,3], Xiao-Lan Wang[1,2,5], Han Jiao[3], Jia Sun[3], Andries Kalsbeek[1,2,4], Chun-Xia Yi[1,2,4 #], Yuanqing Gao[3 #]

Supplementary figures

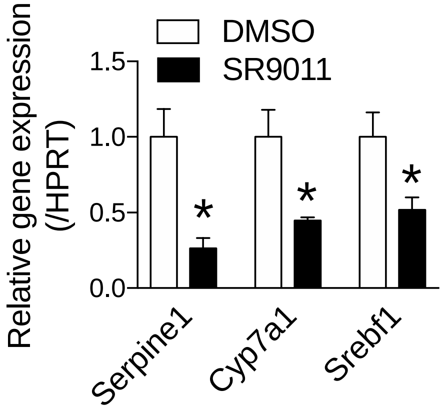

Figure S1. The effects of SR9011 on the Rev-erbα related targets. Primary microglia were treated with SR9011 or DMSO for 24 hours. The Rev-erbα responsive genes *Serpine1*, *Cyp7a1* and *Srebf1* expression is downregulated by SR9011. $p<0.05$* vs DMSO determined by unpaired t-test.

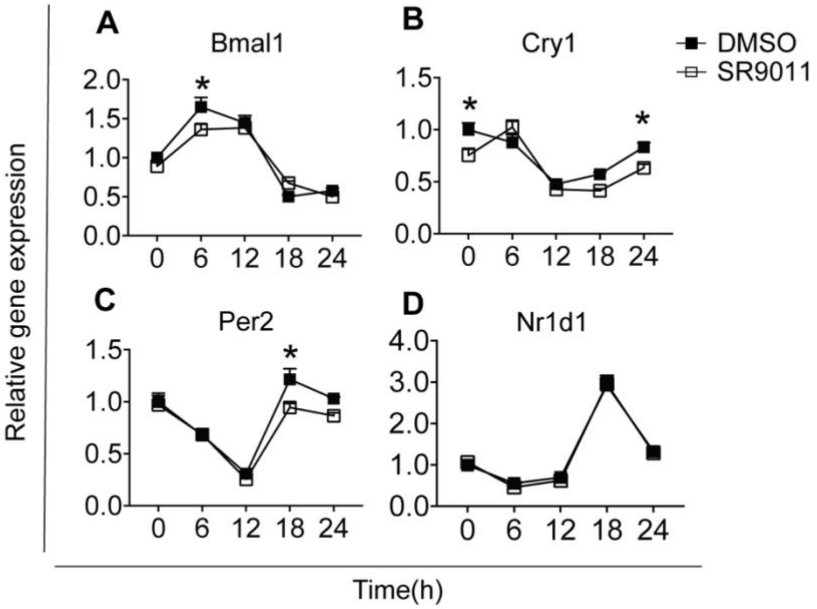

Figure S2. The effects of SR9011 do not persist in the absence of SR9011. Primary microglia were pretreated with SR9011 for 12 hours, synchronized by dexamethasone for 2 hours, and cultured for 0, 6, 12, 18 or 24 hours before being harvested. *Bmal1*, *Cry1*, *Per2* and *Nr1d1* show rhythmic expression in both DMSO and SR9011 treated groups. SR9011 does not disrupt the microglial clock genes rhythmicity when used as a pretreatment (see JTK analysis in Table S3). Data are presented as means ± SEM and statistical significance was determined using Two-Way ANOVA with Bonferroni's post-test and multiple comparison.

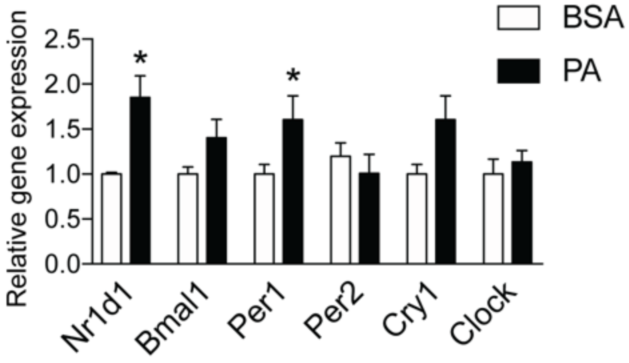

Figure S3. The effect of palmitic acid on core clock genes. Primary microglia were treated with DMSO for 12 hours followed by 50 μM palmitic acid (PA) or BSA treatment for 12 hours (n=4 per group). *Nr1d1* and *Per1* expression increased after 12h incubation with PA compared with BSA. $p<0.05$* vs BSA.

Supplementary tables

Table S1. Primer sequences of target genes.

	**Genes**	**Forward**	**Reverse**	**Product size**	**RefSeq accession**
Clock	*Bmal1*	CCGATGACGAACTGAAACACCT	TGCAGTGTCCGAGGAAGATAGC	215bp	NM_024362.2
	Per1	CGCACTTCGGGAGCTCAAACTTC	GTCCATGGCACAGGGCTCACC	188bp	NM_001034125.1
	Clock	CGATCACAGCCCAACTCCTT	TTGCAGCTTGAGACATCGCT	239bp	NM_001289832.1
	Nr1d1	ACAGCTGACACCACCCAGATC	CATGGGCATAGGTGAAGATTTCT	101bp	NM_001113422.1
	Per2	CACCCTGAAAAGAAAGTGCGA	CAACGCCAAGGAGCTCAAGT	148bp	NM_031678.1
	Cry1	AAGTCATCGTGCGCATTTCA	TCATCATGGTCGTCGGACAGA	196bp	NM_198750.2
Cytokines	*Il1β*	TGTGATGAAAGACGGCACAC	CTTCTTCTTTGGGTATTGTTTGG	70bp	NM_031512.2
	Il6	TTGTTGACAGCCACTGCCTTCCC	TGACAGTGCATCATCGCTGTTCA	198bp	NM_012589.2
	Il10	CGACGCTGTCATCGATTTCTC	CAGTAGATGCCGGGTGGTTC	186bp	NM_012854.2
	Tnfα	ATCGGTCCCAACAAGGAGGA	GCTTGGTGGTTTGCTACGA	137bp	NM_012675.3
	Ccl2	CCACCACTATGCAGGTCTCT	GCATTAACTGCATCTGGCTGAGA	97bp	NM_031530.1
	Gm-csf	TACAAGCAGGGTCTACGGGG	AGTCAGTTTCCGGGGTTGGA	103bp	NM_053852.1
	Tgfβ	ACCGCAACAACGCAATCTATG	CACTCAGGCGTATCAGTGGG	241bp	NM_021578.2
Metabolism	*CD36*	ACAGTTTTGGATCTTTGACGTG	CCTTGGCTAAATAACGAACTCTG	113bp	NM_031561.2
	CD68	TGTTCAGCTCCAAGCCCAAA	GCTCTGATGTCGGTCCTGTTT	196bp	NM_001031638.1
	Cpt1	AGAGCAATAGGTCCCCACTCAA	ATGAAATCACACCCACCACCA	231bp	NM_031559.2
	Pdk1	CGGCGGGGCTCGGTATG	TTCACAAGCATTTACTGACCCGAAG	230bp	NM_053826.2
	Hk2	GGTGAGCCATCGTGGTTAAG	CTTCCGGAACCGCCTAGAAA	295bp	NM_012735.2
	Glut5	TGCCCTATGTCAGCATCGTC	GGACCAAGGCCCACTTGAAT	202bp	NM_031741.1
	Fasn	CTTGGGTGCCGATTACAACC	GCCCTCCCGTACACTCACTC	185bp	NM_017332.1
	Serpine1	GGCACAATCCAACAGAGACAA	CAGTGCCGGGGTAAGAAAGA	182bp	NM_012620.1
	Cyp7a1	GGAATTGCCGTGTTGGTGAG	AGGTTCACCAGCTTTCCTTCT	72bp	NM_012942.2
	Srebf1	CTTGACCGACATCGAAGACAT	GGCATCAAATAGGCCAGGGA	74bp	NM_001276707.1
Housekeeping	*Hprt*	TTGGTCAAGCAGTACAGCCC	CTTGCCGCTGTCTTTTAGGC	227bp	NM_012583.2

Table S2.

Genes	Two-way ANOVA analysis		
	p-value		
	Interaction	Time	SR9011
Bmal1	**0.0055**	**<0.0001**	0.2253
Cry1	**0.0021**	**<0.0001**	**0.0060**
Per2	0.1216	**<0.0001**	**0.0119**
Nr1d1	0.8792	**<0.0001**	0.8006

Table S2. **Two-way ANOVA assessment of effect of Time, SR9011, and Interaction in clock genes in primary microglia with SR9011 before synchronization with dexamethasone for Figure S2.** SR9011, Time and Interaction effects were evaluated in primary microglia for clock genes after treatment with DMSO or SR9011. SR9011 has a significant impact on *Cry1* and *Per2*, and has an interaction effect with time on *Bmal1* and *Cry1*.

Table S3. JTK_Cycle analysis of clock genes in primary microglia pretreated with SR9011 **before synchronization with dexamethasone.**

Gene	DMSO		SR9011	
	P-value	Acrophase	P-value	Acrophase
Bmal1	<0.0001	9	0.002	12
Cry1	<0.0001	3	<0.0001	6
Per2	<0.0001	21	<0.0001	0
Nr1d1	<0.0001	21	<0.0001	21

Table S3. Primary microglia were pretreated with SR9011 for 12 hours, synchronized by dexamethasone for 2 hours, and cultured for 0, 6, 12, 18 or 24 hours before being harvested. Clock genes rhythmicity is analyzed by JTK_Cycle. SR9011 does not disrupt clock genes rhythmicity after being removed from the medium. An estimated shift in acrophase can be found for *Bmal1*, *Cry1*, and *Per2*.
